# Oncolytic Viruses: The Power of Directed Evolution

**DOI:** 10.1155/2012/586389

**Published:** 2011-07-24

**Authors:** Maxine Bauzon, Terry W. Hermiston

**Affiliations:** Bayer Healthcare, Biologics Research, 455 Mission Bay Boulevard South, San Francisco, CA 94158, USA

## Abstract

Attempts at developing oncolytic viruses have been primarily based on rational design. However, this approach has been met with limited success. An alternative approach employs directed evolution as a means of producing highly selective and potent anticancer viruses. In this method, diverse viruses are grown under conditions that maximize diversity and then passaged under conditions meant to mimic those encountered in the human cancer microenvironment. Viruses which evolve to thrive under this selective pressure are isolated and tested to identify those with increased potency (i.e., ability to replicate and spread) and/or an increased therapeutic window (i.e., differentiated replication and spread on tumor versus normal cells), both of which have potential value but the latter of which defines an oncolytic virus. Using ColoAd1, an oncolytic virus derived by this approach as a prototype, we highlight the benefits of directed evolution, discuss methods to “arm” these novel viruses, and introduce techniques for their genetic modulation and control.

## 1. Introduction

As our understanding of cancer increases, the complex nature of this disease which often involves multiple mutations, overlapping signaling pathways, and the ability to adapt and develop resistance to various therapeutics becomes more evident [1–3]. Such a complex disease necessitates equally complex therapies—such as oncolytic viruses [4]. By definition, these viruses infect and selectively replicate in tumor cells resulting in eventual cell lysis. This replication and lysis serves to eradicate the target tumor cells while amplifying the therapeutic in a tumor-dependent fashion, all the while sparing neighboring normal cells. Unfortunately, the promise of oncolytic viruses as agents that selectively find and kill tumor cells has not been fully realized [5–8]. This fact may be due in part to some prejudices taken by researchers in their pursuit of oncolytic viruses. For example, the majority of oncolytic viruses currently studied are Ad5 based primarily, because Ad5 has been widely characterized and methods for its genetic manipulation are well established, making it the practical starting point for most studies. However, there is no clear rationale why Ad5 would make a superior oncolytic virus as opposed to other Ad serotypes or other viral classes. Additionally, the genetic manipulation of today's viruses in an attempt to increase selectivity and or potency may be misguided due to our limited knowledge of the underlying causes and nature of cancer. Therefore, the plasticity and complexity of tumors may hinder the rational design of oncolytic viruses [9]. 

**Figure 1:**
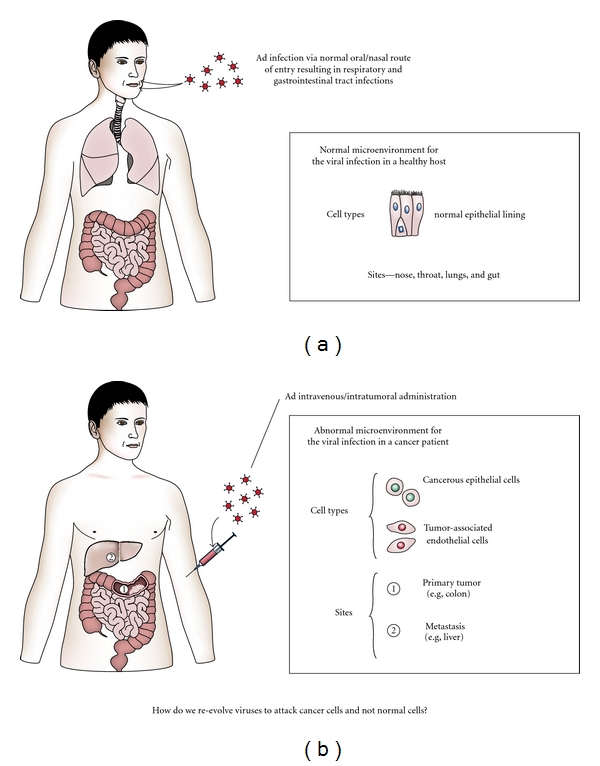
Why we need directed evolution. (a) The starting point of all oncolytic viruses are naturally occurring viruses which infect normal cells they encounter along their standard route of entry. (b) The goal is to develop oncolytic viruses which selectively infect and replicate in cells they would never naturally encounter.

In an attempt to circumvent these issues, researchers are beginning to explore the use of directed evolution as a way to harness the power of natural selection and to derive desirable properties without concern for the mechanism(s) responsible for these properties [10–12]. Directed evolution is not a foreign concept in the field of virology and has been utilized as a way to modulate viral vectors and enhance gene delivery. Such experiments focus primarily on enhancing infectivity or modulating tropism by changes to the viral coat [13, 14]. For oncolytic viruses, the goal is obviously different, namely to drive the viruses to evolve for optimal proliferation in the tumor environment. Normally, viruses infect normal cells. For Adenoviruses, this is via an oral or nasal route of entry and involves the epithelial lining of the nose, throat, and/or gut resulting in a respiratory and/or gastrointestinal infection (Figure 1(a)). As a cancer therapy, the objective is to develop viruses that selectively infect a vastly different set of cells, namely, transformed epithelial cells and tumor associated endothelium located in distinct locations throughout the body, most of which are not normally seen during the typical Adenovirus infection. Researchers are therefore asking a oncolytic virus to efficiently and selectively kill cells to which they would never normally be exposed (Figure 1(b)). The necessary biological alterations needed to reach this goal are complex, but unlike evolution in nature which takes an extended period of time for adaptations resulting in new and desirable traits to accumulate, directed evolution can quickly lead to the rise of novel “species”. Importantly, directed evolution is dependent on 2 factors both of which are completely within the investigator's control, namely, the need for (1)) a diverse starting pool and (2)) selective pressure designed to favor a specific outcome. To maximize starting diversity, the researcher has a myriad of options ranging from a single mutated serotype to entire viral classes. Additionally, the ability of viruses to undergo recombination under certain conditions can increase this starting diversity. Similarly, the directed outcome (increased tumor proliferation) is determined by the selective pressure set by the experimental setup and can be modulated in a number of ways including the source of the tumor cells and growth conditions. 

## 2. ColoAd1

Human Adenovirus is comprised of 56 serotypes which are subdivided into groups A–G. These serotypes differ in a number of ways (e.g., pathology, hemagglutination properties, and cellular receptors used for entry) which help to distinguish them from one another and determine their group affiliation. Despite the diversity of human adenovirus, the majority of all oncolytic viruses are Ad5-based leaving the other serotypes and their oncolytic potential untapped. One way to explore these alternative serotypes is via “directed evolution”. In this approach, Ad serotypes, representing the different Ad subgroups, are pooled and passaged at a high multiplicity of infection on human tumor cell lines representative of the target indication. This process invites recombination and creates selective pressure that gives rise to highly potent viral variants. 

When this approach was applied to the colon cancer cell line, HT29, the result was ColoAd1, the first non-Ad5-based and first directed-evolution-derived oncolytic virus [10]. Chromatographic and sequence analysis revealed that ColoAd1 was Ad11p, a group B virus, with a nearly complete E3 region deletion, a smaller deletion in the E4 region, and a chimeric Ad3/Ad11p E2B region [10, 15]. In vitro studies showed ColoAd1 to be 2-3 logs more potent than either of its parent serotypes (Ad11p and Ad3), the standard Ad5, or the most clinically advanced oncolytic Ad, ONYX-015 [10, 16] Importantly, this increase in potency on cancer cells did not translate to increased potency on normal cells resulting in a therapeutic window that is 3-4 logs greater than Ad5 or Onyx-015. These *in vitro* results were supported by *in vivo *studies in a colon cancer liver seeding xenograft model and *ex vivo* on tumor tissue isolates from colon cancer patients. Parallel studies identified CD46 as a cellular attachment receptor for Ad11p, the parent virus of ColoAd1 [17, 18]. Interestingly, immunohistochemical studies staining for CD46 expression on excised colon cancer material, normal liver tissue and normal colon tissue revealed that strong CD46 staining was consistently seen in colon cancer tissue but was absent or weak in normal colon and liver tissue [10]. This suggests that CD46 expression may be a contributing factor to the observed tumor selectivity of ColoAd1. Importantly, CD46 overexpression has been described for a number of different cancer indications [19, 20], supporting the therapeutic value of this oncolytic virus. Theoretically, CD46 screening of tumor tissue could help guide physicians to identify which patients should be treated with this agent. 

## 3. Manipulation and Control of Novel Viruses

Oncolytic viruses can and have been used to deliver exogenous genetic material, giving them much needed flexibility and complexity as a cancer treatment and augmenting their potential as viable cancer therapies [21]. The ability to engineer the genome of a replicating virus to carry foreign material is referred to as “arming”. This strategy has been used in a variety of ways to alter viral selectivity, potency, safety, and utility [22, 23]. 

Like nonreplicating viruses, early armed oncolytic viruses controlled expression of foreign genes by using an exogenous constitutive or conditional promoter. This approach allowed for high levels of expression and, in the case of conditional promoters, some level of specificity. As the field advanced, researchers demonstrated that expression of exogenous genes could be achieved by using the endogenous expression machinery of the virus itself [24–26]. This latter method proved to have some advantages, the first being that it decreased the size of the foreign DNA that could be inserted into the viral genome. This outcome was beneficial given that viruses have genomic size constraints that greatly effect packaging efficiency. The second advantage of utilizing endogenous expression machinery is that depending on the endogenous promoter used, the timing and level of expression of the foreign gene could be controlled [24–26]. This not only allowed for efficient expression of the exogenous gene but could be used as an added measure of safety. Because the level of expression could be modulated depending on the endogenous promoter used, researchers and clinicians could have greater control over the levels of the armed therapeutic/exogenous gene. Furthermore, by utilizing an endogenous late promoter, it is possible to limit the expression of the gene to late in the viral cycle which, presumably, for an oncolytic virus would never be reached in a nonpermissive cell (normal cell) and, therefore, confine expression only to target cancer cells. Importantly, an armed therapeutic virus can express multiple genes [27] that potentially increase the therapeutic benefit of the virus and/or enable the treating physician with the opportunity to track the virus location (e.g., via imaging [28]) and viability (e.g., through the expression of a protein that can be detected in the blood or is secreted from the treated patient), as a means to better understand when additional treatments are warranted. 

Arming is traditionally carried out by manipulating the viral genome and usually calls for a well characterized system. The novel viruses resulting from directed evolution will make rational insertion of therapeutic moieties extremely challenging, especially when one considers that they would not want to negatively impact the replication capabilities of these viruses which are critical to their clinical benefit. Clearly, innovative approaches will need to be taken in order to manipulate viruses derived in this manner to ensure that this does not occur. One such approach taken uses a transposon-based method to “scan” a viral genome for insertion sites which are compatible with the viral lifecycle [29]. This approach makes it possible to arm these novel viruses with exogenous genes expressed from a foreign promoter or by the inclusion of a splice acceptor from an endogenous promoter [30]. Although this method, like many advances in the oncolytic field, was originally developed on Adenoviruses, it can be extended to any number of viral species whose genome can be cloned into a plasmid. 

Given the potential to derive potent novel viruses from the directed evolution approach, it is very important to keep safety in mind. If these novel viruses are ever to advance as therapeutics, having the ability to control their replication in the treated patient is paramount. To this end, two approaches were taken with ColoAd1. The first was to study the effect on ColoAd1 of two clinically approved antivirals, ribovirin (RBV), and cidofovir (CDV), and the second was to genetically introduce drug sensitivity to the virus by inserting the HSV TK gene into the ColoAd1 genome using the transposon method [31].

Although there are no approved anti-Ad treatments, both RBV and CDV have been used to experimentally treat Adenoviral infections [32–34]. From these studies, RBV was found to be effective on Group C Ads but less so on Group B Ads [35, 36]. Not surprisingly, ColoAd1, being derived from 2 group B viruses was refractory to RBV treatment. CDV has been shown to have better Anti-Ad activity than RBV and when used to treat ColoAd1 was effective at inhibiting viral replication and spread on both tumor and normal cell lines. Interestingly, ColoAd1 was more sensitive to CDV treatment than either of its 2 parental strains, Ad11p or Ad3 suggesting that this increased CDV sensitivity was an outcome of the directed evolution process and not simply an inherited trait [31]. An alternative approach to the use of approved antivirals is the insertion of the HSV TK gene into the viral genome to create sensitivity to the approved drug ganciclovir (GCV) [37]. This approach, made possible by the transposon method of arming was successful in inhibiting ColoAd1 infection of both tumor and normal cells [29, 31]. The potential for using TK expression to track the virus also makes this approach appealing [38]. From these studies, it was demonstrated that ColoAd1 could be controlled through outside intervention. 

The value gleaned from the ColoAd1 experience goes beyond a promising therapeutic, as it validates a new approach for the oncolytic virus field. The directed evolution method that resulted in ColoAd1 could be applied to other cancer types and other viral families. By utilizing this method, researchers are not longer limited to well characterized systems. Moreover, the ease by which viruses are molecularly manipulated need not be the deciding factor for proceeding. Although Adenoviruses have been covered in this paper, this approach is amendable to all viral families and opens up the possibility of harnessing inherent and novel oncolytic properties from a multitude of human viruses. Unlike more deliberately designed approaches, directed evolution capitalizes on the complexity of the tumor and can be directed towards an outcome that depends predominantly on the selective pressure applied by the researcher. Similar to Ad5-based oncolytic viruses, it is possible to arm these novel viruses without interfering with their lifecycle, thus unlocking the potential to modulate their characteristics or utility. Because these viruses are potentially highly potent, developing ways to control their replication should be a priority.
